# Genome-wide search for miRNA-target interactions in *Arabidopsis thaliana *with an integrated approach

**DOI:** 10.1186/1471-2164-13-S3-S3

**Published:** 2012-06-11

**Authors:** Jiandong Ding, Danqing Li, Uwe Ohler, Jihong Guan, Shuigeng Zhou

**Affiliations:** 1 Shanghai Key Lab of Intelligent Information Processing, Fudan University, Shanghai, China; 2 School of Computer Science, Fudan University, Shanghai, China; 3 Institute for Genome Sciences & Policy, Duke University, Durham, North Carolina, USA; 4 Department of Biostatistics & Bioinformatics, Duke University, Durham, North Carolina, USA; 5 Department of Computer Science & Technology, Tongji University, Shanghai, China

## Abstract

**Background:**

MiRNA are about 22nt long small noncoding RNAs that post transcriptionally regulate gene expression in animals, plants and protozoa. Confident identification of MiRNA-Target Interactions (MTI) is vital to understand their function. Currently, several integrated computational programs and databases are available for animal miRNAs, the mechanisms of which are significantly different from plant miRNAs.

**Methods:**

Here we present an integrated MTI prediction and analysis toolkit (imiRTP) for *Arabidopsis thaliana*. It features two important functions: (i) combination of several effective plant miRNA target prediction methods provides a sufficiently large MTI candidate set, and (ii) different filters allow for an efficient selection of potential targets. The modularity of imiRTP enables the prediction of high quality targets on genome-wide scale. Moreover, predicted MTIs can be presented in various ways, which allows for browsing through the putative target sites as well as conducting simple and advanced analyses.

**Results:**

Results show that imiRTP could always find high quality candidates compared with single method by choosing appropriate filter and parameter. And we also reveal that a portion of plant miRNA could bind target genes out of coding region. Based on our results, imiRTP could facilitate the further study of *Arabidopsis *miRNAs in real use. All materials of imiRTP are freely available under a GNU license at (http://admis.fudan.edu.cn/projects/imiRTP.htm).

## Background

MicroRNAs (miRNAs) [1] are a class of 20-nt to 24-nt small non-coding RNA (sncRNA) that has emerged as a key regulator of gene activity. MiRNAs regulate virtually every aspect of biology, including developmental timing, differentiation, proliferation, antiviral defence and metabolism [2]. In plants, miRNAs are processed from larger precursor stem-loops (pre-miRNAs) in the nucleus, mainly by DICER-LIKE1 (DCL1) which excises a double-stranded RNA consisting of a miR and its near-complementary miR* sequence from the other arm of the stem-loop. The miRNA:miRNA* duplex is methylated and translocated to the cytoplasm where it can be loaded into an RNA-induced silencing complex (RISC) that includes a member of the ARGONAUTE (AGO) family as catalytic component. The RISC can then recognize mRNAs containing sequences complementary to the loaded miRNA [3]. In plants, cleavage of the target mRNA is the main mechanism for miRNA action, but there are also direct effects on protein accumulation [4,5], as reported for many animal miRNAs [6,7].

Unlike animals, plant miRNAs generally show a near-perfect complementary target mRNA which immensely facilitates computational predictions [8]. Taking advantage of this property, several methods were developed to search for antisense hits to known miRNAs on *Arabidopsis *mRNAs [9,10]. While both animals and plants rely to a different extent on RNA complementarity to define their targets, some comparable features are employed in target prediction methods/tools for both animal and plant miRNAs. Notably, RNAhybrid [11] was first developed to identify miRNA-Target duplexes in *D. melanogaster, D. pseudoobscura*, and *A. gambiae*, and was more recently adapted to the specific requirements of plant miRNA target prediction, with outstanding results in *Arabidopsis thaliana *[12,13].

Features reported for plant miRNA-target interactions in previous studies can be divided into three categories: (i) duplex pairing, with specific consideration of the seed region (2-8nt) in particular for animals, and the central region in plants (9-11nt), (ii) evolutionary conservation of MTI sites, and (iii) MTI site accessibility.

**Figure 1:**
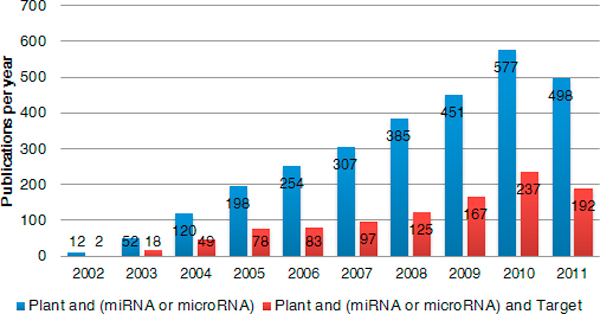
**Growth of plant 'miRNA' and 'miRNA Target' in PubMed**. In last decade, miRNA changed to be a hot research topic. It's impossible to establish the exact number of how many labs are studying this small molecule and how much funding is spent. However, the number of relevant papers can broadly reflect this trend. We then collect plant miRNA related articles indexed by PubMed (collected on Jun.13th, 2011). Statistical results show that these articles grow in an exponential way.

While experimental studies have already identified a certain amount of MTIs, this issue is far from resolved. On one hand, plant miRNA and miRNA-target related research increased yearly during the last decade (Figure 1). The list of known miRNAs is large and increasing rapidly [14,15]. In the latest miRBase (Release 17.0), over 3,000 plant miRNAs are registered. On the other hand, only a few miRNA-target interactions are experimentally validated; thus, predicting and validating miRNA targets is one of the key topics in understanding miRNA biology. Although many target prediction methods/tools have been developed, several new discoveries are still worth considering.

First, the effect of different seed site matches has been evaluated by different means. In animals, this led to the definition of several canonical seed types that differ in abundance and downstream effect [7]. Recent studies suggested that the majority of functional target sites are formed by less specific seeds of only 6 nt indicating a crucial role of this type, and they also suggest that the majority of functional sites remain uncovered by common prediction methods [16]. Second, not all animal targets are defined by 5' seed matches, but other types of complementarity, including a small number of near-perfect target sites inducing cleavage [17]. This feature was considered in a target prediction method for human/mouse miRNAs [18]. Third, increasing numbers of reports emphasize the importance of multiplicity of target sites in *Arabidopsis*. As example, some primary and secondary trans-acting siRNAs are generated from regions of *Arabidopsis *genes with two or more known miRNA/siRNA complementary sites [19,20]. Fourth, miRNA-target interaction is determined by multiple factors. Considering the extent of influence of each of these factors in recognition mechanisms is still unclear to date, and current predictive approaches are often based on only some factors [21]. As result, our study clearly shows that current methods can give different predictions on identical miRNA/mRNA sets (see Results).

All the problems mentioned above exist in predicting miRNA-target interactions of both animal and plant miRNAs. One successful attempt to address these is by integrating diverse approaches and datasets in a comprehensive manner that may substantially improve animal MTIs prediction. To date, miRNAmap 2.0 [22], miRecords [23], miRGator 2.0 [24], miRGene 2.0 [25], miRror [26] provide miRNA targets by integrating extensively adopted target prediction methods. Moreover, Tarbase [27], miRDB [28], miR2Disease [29] and miRTarBase [30] are established to provide experimentally validated MTIs.

Here, we present the first integrated MTI identification toolkit--imiRTP (integrated miRNA Target Prediction) for *Arabidopsis thaliana*, the most studied plant model species. It integrates 4 powerful predictors based on different factors, and is evaluated on 142 experimentally validated MTIs and 25,688 MTIs predicted by CleaveLand [31] on several Degradome data sets. Besides integrating different prediction methods and data sources, imiRTP also offers 4 effective filters to select high quality MTIs and supports diverse outputs to facilitate further analysis.

## Methods

**Figure 2:**
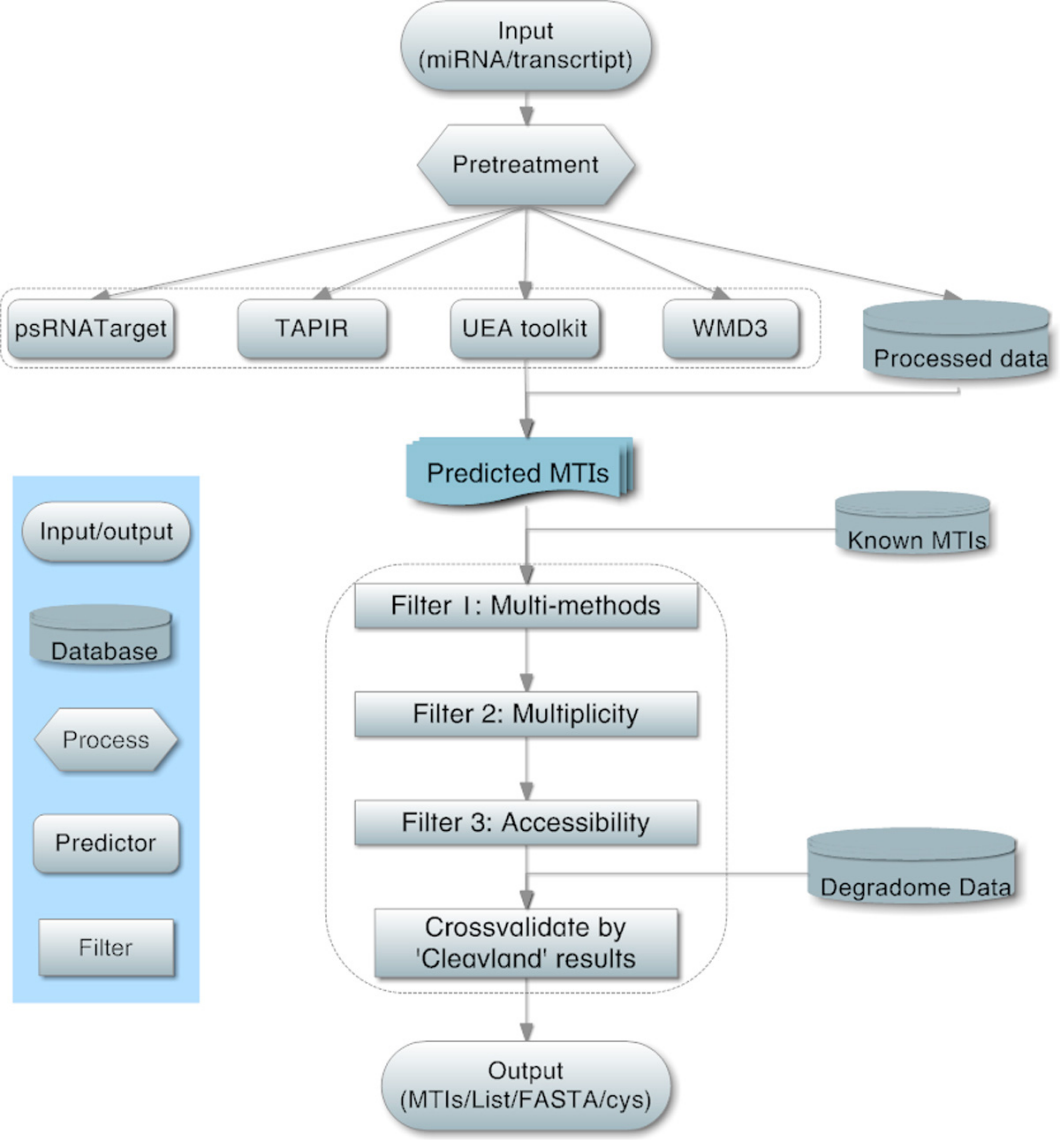
**The pipeline of imiRTP**. Here we present the first integrated miRNA Target Prediction toolkit (imiRTP) to search MTIs for *Arabidopsis*. In this toolkit, four online plant miRNA target prediction tools are considered. We then employ four local filters to select high quality candidates. Finally, it could output predicted results in several different formats. As for data, we collect 142 experimentally validated *Arabidopsis *MTIs and also 25,688 Degradome-seq support *Arabidopsis *MTIs.

Over the last decades, studying the biogenesis and function of miRNA has been an important task. Here, we integrate several plant miRNA target prediction methods and collect data from various sources, aiming to effectively identify *Arabidopsis thaliana *MTIs on the genome-wide scale. Details of the imiRTP pipeline are shown in Figure 2.

Considering target prediction is extremely computation time consuming, imiRTP submits input miRNA and/or transcript sequences to corresponding online predictors and then collects results for local analysis to save time. Moreover, data will be processed before submission with the aim to further save time. First, all input miRNAs are searched against a pre-processed miRNA dataset, consisting of all *Arabidopsis *miRNAs in miRBase (Release 17). Matched miRNA will not be submitted to online predictors; instead, their MTIs will be searched from already computed prepared genome-wide results. Second, MIR genes contained in transcript sequences will be removed. Then, all online collected and locally searched MTIs are combined together. In post-processing, imiRTP provides four filters to narrow results, thus substantially improving the predictions.

### Dataset

We downloaded all 255 *Arabidopsis thaliana *miRNAs arising from 231 pre-miRNAs in the miRNA database miRBase [32] (http://www.mirbase.org/). Transcript sequences (CDS, 5'UTR and 3'UTR) were downloaded from the central database TAIR [33] (http://www.arabidopsis.org/, Release 9).

A previous study [13] has collected 102 experimentally validated *Arabidopsis *MTIs from several publications [34-36], and these MTIs have been used as benchmark in comparison [13,37]. As the most complete online experimentally validated MTI database, miRTarBase has accumulated nearly 4,000 MTIs by manually surveying over 1,100 pertinent publications after systematic text mining to filter research articles related to functional studies of miRNAs [30]. Sixty two experimentally validated *Arabidopsis thaliana *MTIs were downloaded from miRTarBase (http://mirtarbase.mbc.nctu.edu.tw/, Release 2.4). After removing duplicates, a total of 142 experimentally validated MTIs were collected.

Recently, high-throughput CLIP-Seq (HITS-CLIP [38], PAR-CLIP [39]) and Degradome-seq [34,36] methods have been applied to identify the sites of Argonaute interaction and miRNA cleavage, respectively. 25,688 degradome sequencing supported *Arabidopsis thaliana *MTIs were downloaded from starBase [40] (http://starbase.sysu.edu.cn/, Release 2.0). These MTIs are predicted by CleaveLand (version 2.0) [31] with a cutoff of 7.

### Online predictors

A number of algorithms and tools have been developed to predict complementarity between miRNAs and their targets (Table 1). But the types of methods applied, the input miRNA and transcript sequences used and the performance evaluation vary widely between tools. In this work, we prudentially chose four existing predictors to construct the core component of imiRTP's first stage. All of them rely on different combinations of seed pairing, central pairing, and hybridization energy of target site.

**Table 1:** Summary of target prediction tools for plant miRNA

Method	Link	**AUTS**^**1**^	**Limit**^**2**^	**Spe**^**3**^	Ref
miRU	N/A	N	1 miR	N	[57]
**psRNATarget**	http://plantgrn.noble.org/psRNATarget/?function=3	Y	20 M/200 M	EH	[45]
**TAPIR**	http://bioinformatics.psb.ugent.be/webtools/tapir/	Y	50 kb/40 M	H	[13]
**UEA_sRNA**	http://srna-tools.cmp.uea.ac.uk/plant/cgi-bin/srna-tools.cgi?rm=input_form&tool=target	N	50 miRs	S	[42]
**WMD3**	http://wmd3.weigelworld.org/cgi-bin/webapp.cgi?page=TargetSearch;project=stdwmd	N	1 miR	H	[41]
TargetAlign	http://www.leonxie.com/targetAlign.php#	Y	locale	ES	[37]
Targetfinder	http://jcclab.science.oregonstate.edu/node/view/56334	Y	locale	N	[58]

^1 ^AUTS - Accepts User-supplied Transcript Sequences.

^2 ^Input limitations. Limitations for miRNA and transcript input are on the left and right sides of the slash.

^3 ^Running speed at the time of testing. E-Extremely, H-High (≤ 5 min), N-Normal (5-30 min), S-Slow (≥ 30 min).

WMD3 [41] is based on principles of artificial miRNAs, which support the notion that extensive base pairing with targets is required for plant miRNA function. It predicts targets using previously determined parameters of target selection for natural miRNAs. The number of mismatches (cutoff 5) and hybridization energy ratio (≥ 70%) are two critical parameters when searching targets. In this method, a GU wobble pair is counted as 1 mismatch.

UEA_sRNA is included in the UEA toolkit [42] which identifies plant sRNA (miRNA/siRNA) targets. The rules used for target prediction are based on factors suggested in previous studies [35,43]. Both seed region and central region are considered. Mismatches in the central region (9-11nt) are not allowed. The hybridization energy ratio is computed as the MFE (minimum free energy) of miRNA:miRNA* instead of the traditional optimal energy that is calculated by the miRNA and its perfect reverse complement. Considering the similar rules used in UEA_sRNA and Targetfinder [44], the latter is not integrated in imiRTP.

TAPIR offers potential plant miRNA targets using a fast (FASTA) search engine and a precise (RNAhybrid) engine. A miRNA target score is modified from a previous study [35]. Mismatch, gap and wobble pairs inside and outside the core region (2-12nt) are counted differently. Again, the hybridization energy ratio is considered (≥ 70%). Considering a previous assessment [13], we chose to integrate the fast FASTA algorithm. Another Smith-Waterman-like alignment, Target-align, has been shown to perform better than TAPIR [37]. However it is not practical for prediction of MTIs on genome-wide scale since too many parameters are considered.

psRNATarget [45] is designed for plant sRNA target prediction with an efficient distributed computing back-end pipeline that runs on a Linux cluster. This tool can rapidly search for potential MTIs and it is the first to provide multiplicity information and functional type (cleavage or translation) determined by the occurrence of a mismatch in central region (9-11nt). Unfortunately, the multiplicity is only reported but not considered in prediction.

In Table 1, we list the input limitations of existing online predictors. Here, the third mission of the pre-processing procedure is rearranging user-submitted files, in order to guarantee flexibility when working with imiRTP.

### Local filters

The secondary stage of imiRTP includes several effective filters. The user can remove predicted MTIs with one filter or various ensembles. When comparing performance among predictors, there are usually two levels: mRNA level and target-site level. Here, we use the latter. Putative target sites that overlap at least 90% will be grouped into one common MTI.

#### Multi-method

MTI predicted by single method is usually not as credible as those identified by multiple methods. Keep this in mind, we introduce the multi-method filter to help user to select multiple predicted MTIs.

#### Multiplicity

Strong miRNA targets tend to have multiple target sites instead of one single site [46]. Considering the number of putative miRNA site per mRNA can therefore significantly enhance MTI prediction. In reported plant miRNA target prediction tools, the importance of the target site multiplicity was generally underestimated. Default cutoff of multiplicity is 2.

#### Accessibility

The frequently considered free energy of interaction of a miRNA and its target is generally not a very good predictor [21]. An effective MTI needs an open structure on the target site to begin the hybridization reaction, an issue which has been extensively explored in animals [47-49]. The RNAup program in Vienna package is used to calculate secondary structure in target site regions [50]. The RNAup takes into account the hybridization energy and the free energy needed to open the target site, which turns out to be the real accessibility. The default threshold of accessibility is set to the highest value that observed for all 125 validated MTIs recovered by imiRTP.

#### Degradome-seq support

Degradome-seq (also known as PARE and GMUCT) directly sequences degradome tags derived from the 5' ends of uncapped mRNAs and delivers an empirical overview of cleaved sRNA targets without computational predictions or overexpression. In this work, 25,688 Degradome-seq results were collected from [40], which were then used to filter computationally predicted MTIs at the mRNA level. The default cutoff 4.5 is suggested by [31].

### Input and output

The imiRTP toolkit accepts user-submitted miRNA and/or transcript sequences for analysis, i.e. (i) searching user-submitted miRNAs against included TAIR transcripts; and (ii) searching user-submitted miRNAs and user-submitted transcripts. After all files are successfully submitted, imiRTP will search targets based on selected predictors and combine all results into one group.

Once the submitted analysis is completed, imiRTP outputs details of predicted MTIs to one file and outputs statistics of every unique miRNA-target (mRNA level) predicted by which predictor to another file. A sort tool is implemented to easily browse through the results. In addition, imiRTP allows users to extract several essential columns from results related to single miRNAs or mRNAs, which greatly facilitate further analysis, i.e. motif discovery, SNP detection and regulatory network analysis.

### Implementation

imiRTP was implemented in C++. Online interaction programs were implemented in C# and java. Both are tested on Windows platform. Computation of the accessibility profiles in the post-processing steps is performed with the help of RNAup program. When calculating the accessibility, imiRTP extracts a maximum of 100nt flanking sequences on both upstream and downstream of a target site instead of using the whole sequence, thus greatly reduces the calculating time.

## Results

To test the toolkit, we evaluated imiRTP on a reference set with 142 experimentally validated mRNA level MTIs [13,40] (Additional file 1). All comparisons are executed at the target-site level, except Degradome-seq support. The numbers of validated MTIs listed in Table 2 are therefore always larger than 142. To facilitate the comparison, we defined and considered several criteria:

**Table 2:** Performance of four predictors*

Method	**PM**^**†**^	**VM**^**†**^	**CM**^**†**^	AP^#^(%)	TP^#^(%)
psRNATarget	541	366	100	32.35	70.42
**TAPIR_FASTA**	622	411	116	33.92	**81.69**
UEA_sRNA	362	269	110	**25.69**	77.46
WMD3	615	411	98	33.17	69.01

Average	535	364	106	31.28	76.41

^† ^PM - predicted MTI; VM - experimentally validated MTI; CM - collapsed MTI.

^# ^AP - additional prediction; TP - true positive.

* Results achieved with default parameters.

1) **Additional prediction **Percentage of predicted target-site level MTIs that do not belong to the reference set. Smaller is better.

2) **True positive **Percentage of MTIs that each predictor or predictor ensembles can recover from the reference set. Since all validated MTIs are on mRNA level, predicted target-site level MTIs are first collapsed to mRNA level. Larger is better.

3) **Filter power **Geometric mean of filter ratio and additional prediction. Filter ratio is defined as the fraction of predicted MTIs that are filtered by a given filter compared to the number of total predictions. Smaller is better.

### Performance of online predictors

First, we tested the four selected predictors with the reference set and compiled the results in Table 2. At default settings TAPIR_FASTA identifies the most validated MTIs (116/142), while WMD3 identifies the least (90/142). This demonstrates the specific importance of the core region (2-12nt), which covers both the seed region and central region. UEA_sRNA gets the lowest additional prediction with only 362 predicted MTIs, which is considerably smaller than other three methods. For one reason, UEA_sRNA uses stringent parameters. The other reason might be the special hybridize energy ratio computed by this method.

**Figure 3:**
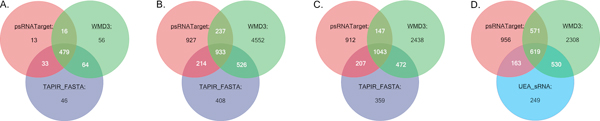
**The degree of overlap between the four MTI prediction tools at target-site level and mRNA level**. In order to directly compare different online predictors, we run them on a conducted reference set and the whole *Arabidopsis Thaliana *cDNA sequences. Results turn to be that different predictors could give different results. (A.) Target site level predictions on reference set. (B.) Target site level predictions on whole cDNA sequences. (C.) mRNA level predictions on whole cDNA sequences. (D.) mRNA level predictions on whole cDNA sequences.

Moreover, on the reference set, different methods lead to different results at the target-site level and mRNA level. The percentage of common MTIs that predicted by any two predictors lies between 42.14% and 83.25% (Additional file 2, Table S1). We first selected three methods (psRNATarget, TAPIR_FASTA and WMD3) and show their results in Figure 3A, since these three methods give the most similar predictions. However, the fraction of highest credible MTIs, those that are predicted by all three methods, is still lower than 50% both at the target-site level (Figure 3B) and mRNA level (Figure 3C) on the genome-wide scale. Indeed, the results change very little at different levels, except WMD3, for which 2,240 duplicate MTIs are removed at the mRNA level. A certain number of targets are identified by only one method, as a result of the various factors considered by different methods (Figure 3B-D). A similar fraction of common MTIs are identified by TAPIR/psRNATarget and TAPIR/UEA_sRNA on the genome-wide scale, while psRNATarget and UEA_sRNA share fewer common predictions. One likely reason might be the opposite decision made on mismatches in the central region (9-11nt). Another reason might be that psRNATarget does not pay specific attention to the seed region as UEA_sRNA does. WMD3 leads to the highest number of predictions, as it does not consider any specific regions (like seed or central region). Yet, it identifies only 98 validated MTIs. All these observations show that it is necessary to develop a more general and accurate method to search qualified targets of plant miRNA through combining various sources.

### Performance of local filters

**Figure 4:**
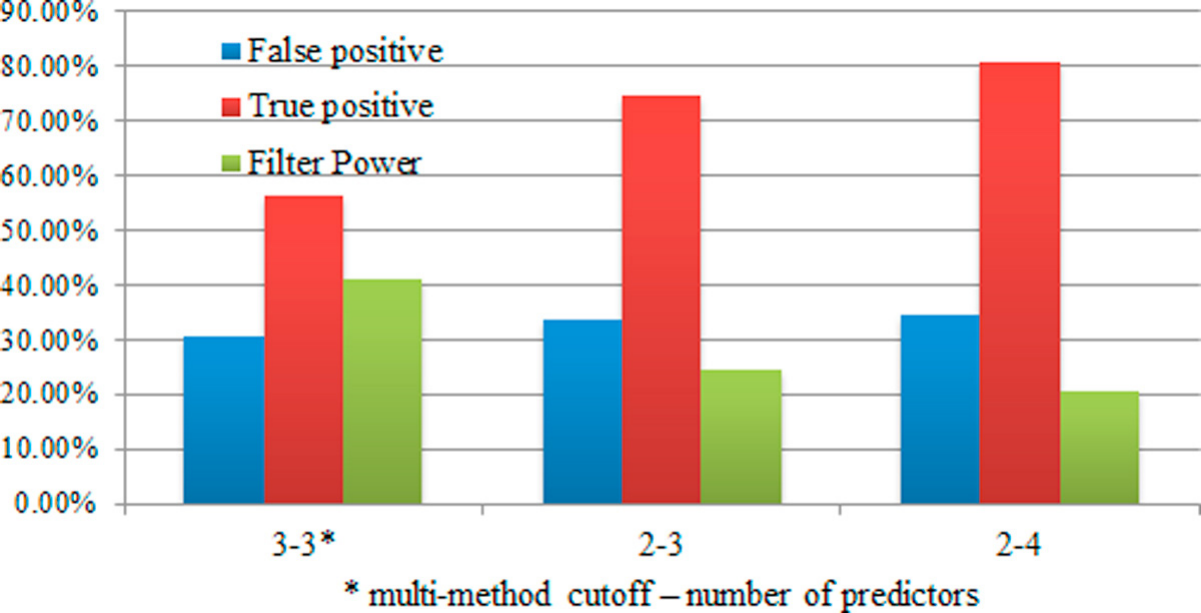
**Performance of imiRTP by integrating multiple predictors**. It's clear that directly combine results of single online predictors, we can't expect imiRTP achieves higher true positive than the best single method. At the other side, we will get the lowest additional prediction, which contains fewest true negatives. Here, with the multi-method cutoff, imiRTP can achieve the best balance between true positive and additional prediction.

Direct combination of different predictors can lead to fewer additional predictions, but also can greatly decrease the number of true positives (Additional file 2, Table S2). imiRTP therefore accepts user-defined cutoff to meet different needs; e.g. when 3 predictors are chosen, the user can use a loose cutoff, like 2, to select more predicted results. Results show that with more predictors and looser cutoff, additional predictions increase slowly, while true positives increase and the filter power decreases rapidly (Figure 4). Specifically, with a combination of 4 predictors and the multi-method cutoff 2, imiRTP identifies only one experimentally validated MTI less than TAIR_FASTA but 9 more high quality predicted MTIs.

We next compared the effect of other filters. To achieve reliable results, we constructed a benchmark set by selecting several best combinations of predictors and/or multi-method cutoff from the different groups mentioned above (highlighted in Table 2, and in Additional file 2, Tables S1 & S2).

**Figure 5:**
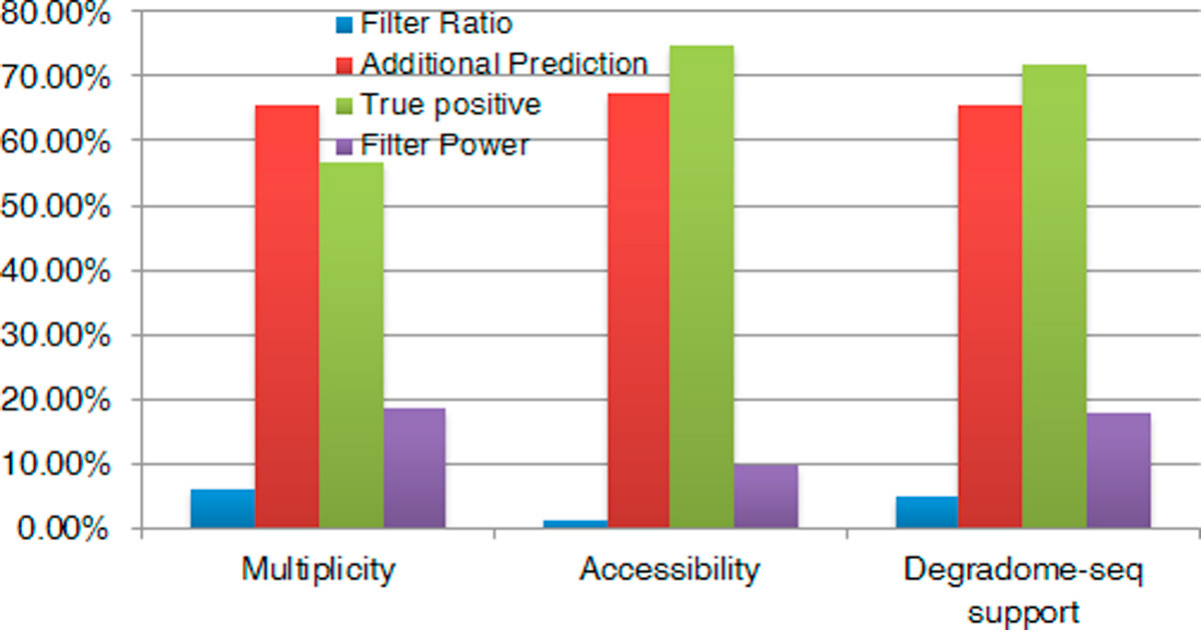
**Performances of three locale filters**. Based on the benchmark, we also compare other three locale filters. Results shown here are achieved by best cutoffs (multiplicity: 2, accessibility: -15.87 kal/mol, Degradome-seq support: 4.5).

Based on this benchmark, we tested the other three filters: multiplicity, accessibility and Degradome-seq support (Figure 5). We first find that multiplicity (cutoff 2) and Degradome-seq support (cutoff 4.5) get similar performances. However, more validated MTIs are removed by multiplicity, which indicates that in *Arabidopsis*, maybe one fifth of target genes contain only one unique functional miRNA site (Additional file 2, Tables S3 & S4).

As for accessibility, the default cutoff for RNAup program is -15.87 kcal/mol, which is the maximum value of 119 out of all 125 validated MTIs that could be recovered by imiRTP. Thermodynamic related features are considered by all four online predictors but with different calculation methods. For example, the RNAup program has been employed by psRNATarget, while RNAcofold was set as default in WMD3, and the flanking regions around the target site are different. Here, we employ RNAup to compute the accessibility uniformly. Because of the specific cutoff (-15.87 kcal/mol), only 6 validated MTIs, whose accessibilities couldn't be calculated by RNAup, are removed. Correspondingly, it results in the highest true positives. On the other hand, the filter ratio is extremely low (Additional file 2, Table S5), which then gives rise to contain a little bit more additional predictions. When validating the effectiveness of the three local filters, results also show that the combinations of multiple methods always achieve similar or better predictions than any single method.

### Performance on whole transcripts

Plant miRNA binding sites occur typically in the coding region of target genes [51], whereas in animals, they are most often found within the 3'UTR [52]. However, new findings indicate that both animal and plant miRNAs can target 5'UTR, 3'UTR and coding regions [53,54]. We therefore run the programs specifically on *Arabidopsis thaliana *CDS, 5'UTR and 3'UTR sequences downloaded from TAIR (version 9).

Because only psRNATarget and TAPIR accept user-submitted transcript files, only these two predictors are considered here. Consistent with previous studies, 74.63% (897/1202) miRNA target sites identified by imiRTP falls within the coding region. 15.46% miRNAs (32/207) that come from 5 families are predicted to target mRNAs in the 3'UTR. Only 4 miRNAs belonging to 2 families (MIR399 and MIR827_3) can bind to the 5'UTR region. Results are shown in Table 3. The statistics of MTIs that predicted by different methods and filters are compiled in Additional file 2, Table S6-S12. Considering that secondary structure plays less of an importance in coding region than in the UTRs, accessibility here is less effective as in animal target prediction (Figure 5).

**Table 3:** Genome-wide results*

	5' UTR	Coding region	3' UTR
Predicted MTI^1^	52	897	253
Validated MTI^2^	5	254	81
Collapsed MTI^3^	2	80	10

^1 ^Common MTIs predicted by psRNATarget and TAPIR.

^2 ^Number of validated MTIs on target site level.

^3 ^Number of validated MTIs on mRNA level.

* All MIR genes are removed.

## Discussion

In order to verify the effectiveness of the imiRTP toolkit, we collected 142 experimentally validated MTIs from previous studies within a reference set. We find that four online predictors integrated within imiRTP give highly different target-site level results (Additional file 2, Table S1), especially on the genome-wide scale (Additional file 2, Table S9).

Degradome-seq is a novel technology that is independent of computational methods. However, if we directly compare the results of imiRTP and CleaveLand at the target-site level, the fraction of common MTIs decreases dramatically from ~50% to ~2% (data not shown). Moreover, even the combination of all four predictors could only identify 88.03% (125/142) validated MTIs.

Besides psRNATarget, all existing target prediction methods and Degradome-seq for plant miRNA are looking for features specific to target cleavage. As a consequence, a portion of additional predictions might in fact be true positive, although many of them are indeed false positives.

All these results indicate the importance of integrating multiple methods and the introduction of translational repression related factors. Additionally, our results have indicated that proper filters can efficiently identify potential MTIs from large candidate sets.

Future work mainly includes the following parts. (i) Integrate additional existing and novel target prediction methods, (ii) support more plant species, (iii) improve and consider other efficient filters, like computing the accessibility with Raccess [55] and RNAplex [56] and selection of predicted MTIs by evolutionary conservation, (iv) maintain imiRTP with future experimentally and Degradome-seq validated MTIs.

## Conclusions

In this work, we propose the first integrated miRNA target prediction toolkit for *Arabidopsis thaliana*. The imiRTP toolkit brings new features compared to existing methods. The ability to use different predictors and filters to search qualified MTIs, the rich output results, and the use pre-computed results should make imiRTP a useful and efficient resource for the plant research community.

## Competing interests

The authors declare that they have no competing interests.

## Authors' contributions

JD designed the pipeline, wrote the code and prepared the manuscript. DL helped to write the code. UO, JG and SZ helped to prepare the manuscript. All authors read and approve the final version.

## Supplementary Material

Additional file 1 **All 142 experimentally validated *Arabidopsis thaliana *miRNA target interactions**. These MTIs are collected from previous studies and an existing database. Click here for file

Additional file 2 **All 12 additional tables are compiled into one file**. These tables give more details of figures shown in this article and support our conclusions. Click here for file
